# Polymorphisms in Toll-Like Receptor 10 and Tuberculosis Susceptibility: Evidence from Three Independent Series

**DOI:** 10.3389/fimmu.2018.00309

**Published:** 2018-02-23

**Authors:** Yu Wang, Miao-Miao Zhang, Wei-Wei Huang, Shou-Quan Wu, Ming-Gui Wang, Xiao-Yan Tang, Andrew J. Sandford, Jian-Qing He

**Affiliations:** ^1^Department of Respiratory and Critical Care Medicine, West China Hospital, Sichuan University, Chengdu, China; ^2^Centre for Heart Lung Innovation, University of British Columbia and St. Paul’s Hospital, Vancouver, BC, Canada

**Keywords:** TLR10, tuberculosis, latent tuberculosis infection, polymorphisms, susceptibility

## Abstract

**Background:**

The toll-like receptor 2 (TLR2)-mediated immune response is critical for host defense against *Mycobacterium tuberculosis*. There is evidence that TLR10, a TLR2 signaling modulator, may be involved in progression of tuberculosis (TB).

**Methods:**

Using a self-validating case–control design, we tested for an association between seven *TLR10* polymorphisms and susceptibility to TB in three independent series with two distinct populations. Single-nucleotide polymorphism (SNP) genotypes were determined by the SNPscan^TM^ method. Three genetic models (additive, dominant, and recessive) as well as multiple-SNP score analyses were used to evaluate the risk of TB associated with the *TLR10* SNPs.

**Results:**

By comparing TB patients with healthy controls, we observed two SNPs (rs11466617 and rs4129009) that were associated with decreased risk of TB in the Tibetan population, but did not in the Chinese Han population. Further analysis demonstrated that the rs11466617 Chengdu cohort genotype served as a protective factor against the progression of latent TB infection (LTBI) to active TB under the recessive model. None of the SNPs were significantly different in comparisons of TB-uninfected people with LTBI individuals. Additionally, when the underlying four TB-associated loci were considered together in a multiple-SNP score analysis, we observed an allele dose-dependent decrease in TB risk in Tibetans.

**Conclusion:**

Variants of *TLR10* may show an ethnic specificity on susceptibility to TB in Tibetan individuals. rs11466617 affected the susceptibility to progress from LTBI to active TB disease, but was not associated with the establishment of LTBI after *M. tuberculosis* exposure. More studies are needed to verify this genetic epidemiological result and unravel the role of *TLR10* SNPs in the pathogenesis of TB.

## Introduction

Tuberculosis (TB), a chronic pulmonary disease caused by *Mycobacterium tuberculosis* (Mtb) infection, remains a leading global health threat with an estimated 10.4 million incident cases and 1.8 million deaths worldwide in 2015 (1). It is estimated that one third of the world’s population is infected with Mtb, yet only approximately 5–15% of infected individuals develop active TB and the rest remain asymptomatic, which is termed latent TB infection (LTBI) (2). A complex interaction occurs between Mtb and the host immune system, which begins with the inhalation of Mtb-containing aerosol and ultimately leads to the replication of Mtb. This pathway results in tissue inflammation and damage that trigger clinical symptoms (3). Patients who are immunocompromised due to HIV coinfection or diabetes are predisposed to active TB (4), emphasizing the important role of host immunity in the progression of this disease. In addition to environmental risk factors, genetic polymorphisms can influence the expression and/or activity of proteins involved in host immunity, which may result in susceptibility to TB and, to some extent, explain the TB patients without identifiable risk factors (5, 6). Numerous candidate genes have been associated with risk of TB, including members of the toll-like receptor (*TLR*) family (*TLR1, TLR2, TLR4, TLR6, TLR9, TLR10, TIRAP*) (7–9), which are widely expressed on various immune cells including monocytes, macrophages, dendritic cells, and lymphocytes and may modulate activation *via* TLR ligands (10, 11).

TLR10 is a TLR family member first discovered by Chuang et al. (12), which has 50 and 49% amino-acid sequence homology with TLR1 and TLR6, respectively. TLR10 remains an orphan receptor without a confirmed ligand, signaling pathway, or biological function. However, it is widely expressed in lymphoid tissues including the spleen, lymph node, thymus, and tonsils as well as a number of leukocyte subtypes, especially B cells (13). This expression pattern suggests that TLR10 has an immune function similar to other TLRs. Recently, a number of studies indicated that TLR10 serves as a modulatory pattern-recognition receptor with mainly inhibitory properties on TLR2-derived immune responses (14, 15), which are involved in the progression of TB (16, 17).

A previous study reported an association between *TLR10* single-nucleotide polymorphisms (SNPs) and risk of TB in humans (18). However, SNPs located in non-coding regions, which may affect expression of the *TLR10* gene and thus influence disease susceptibility (19), were not investigated. It is also unclear whether these SNPs could mediate progression of LTBI to active TB. Therefore, to give a comprehensive view of the association between *TLR10* SNPs and risk of TB, we performed a self-validating association study based on three independent case–control series.

## Materials and Methods

### Study Design and Subjects

All protocols for this study were reviewed and approved by the Institutional Review Board of the West China Hospital of Sichuan University in 2013 (Protocol no. 131). We evaluated associations between *TLR10* SNPs and TB by analyzing the genotype distributions of cases and controls in three independent series: Aba Tibetan Autonomous Prefecture cohort (ATAPC), Chengdu cohort (CC), and Chengdu LTBI cohort (CLC). Subjects in ATAPC and CC consist of two subgroups: TB patient group and healthy control (HC) group. A total of 1,216 self-identified Tibetan individuals (613 TB patients and 603 controls) in ATAPC were enrolled from the People’s Hospital of Aba Tibetan Autonomous Prefecture, located in northern Sichuan province, China, which is a Tibetan-predominant region. In addition, 1,185 Chinese Han subjects (580 TB patients and 605 controls) in CC were selected from our previous TB genetic association study conducted at the West China Hospital in Chengdu, Sichuan, China (20). The diagnosis of TB was based on the findings from Mtb, clinical symptoms, radiological evidence, and the response to anti-TB treatment, as described previously (20). The control groups in ATAPC and CC consisted of unrelated healthy individuals having routine-scheduled physical exams in the same region with no history of TB, who were gender and age matched to the cases.

We also performed a case–control study in CLC, which included three subgroups: 209 TB patients, 200 LTBI individuals, and 204 HCs, to investigate whether the *TLR10* SNPs were associated with Mtb infection and/or the progression of LTBI to active TB. As with CC, the subjects in CLC were recruited from the West China Hospital in Chengdu. TB patients in this study were bacteriology-confirmed cases having positive of culture and/or acid-fast bacilli (AFB) and/or polymerase chain reaction of Mtb DNA in sputum or body sites, with a typical clinical complaint, e.g., fever, cough, sweating, hemoptysis, or weight loss. Both LTBI and HC subjects were unrelated and asymptomatic contacts of TB patients with normal radiological manifestations and negative sputum smear and culture. A contact was defined as someone who shared the same airspace with a sputum-positive TB patient for at least 15 h per week for >1 week or a total of 180 h during the date of onset of cough until 2 weeks after the initiation of appropriate anti-TB treatment (21). LTBI was defined as a positive result for the QuantiFERON-TB Gold in-tube (QFT-GIT) assay and HC was defined as a negative result for the same assay. Individuals demonstrating the presence of coinfection with HIV, cancer, diabetes, immune system diseases, chronic respiratory diseases, or on steroids for inflammatory conditions were excluded from this study. Written informed consent was obtained from all the study participants. The inclusion and exclusion criteria of each study are detailed in Table S1 in Supplementary Material.

### Single-Nucleotide Polymorphism Selection

Single-nucleotide polymorphisms located within the region 3,000 base pairs upstream and 300 base pairs downstream of *TLR10* in Chinese Han in Beijing were obtained from the International HapMap Project (accessed on May 1, 2016).^1^ We used Haploview 4.2 (Broad Institute of MIT and Harvard, Boston, MA, USA) to capture tagSNPs using common genetic variation [minor allele frequency (MAF) ≥5%] with strong coverage [linkage disequilibrium (LD) *r*^2^ ≥ 0.8]. Seven tagSNPs (rs10004195, rs11096956, rs11466617, rs11466655, rs4129009, rs11096957, and rs11466653) were retained for analysis in this study.

### DNA Isolation and Genotyping

Genomic DNA was extracted from peripheral venous blood using the AxyPrep DNA Blood kit (Axygen Scientific Inc., Union City, CA, USA). The tagSNP genotyping was implemented using a custom-by-design 2 × 48-Plex SNPscan™ Kit (cat. #G0104, Genesky Biotechnologies Inc., Shanghai, China), which was based on double-ligation and multiplex fluorescence polymerase chain reaction (22). The details of the genotyping assays are described in the Supplementary Materials. Quality control measures included blinded repeat genotyping of 5% of the samples for each SNP.

### Interferon-γ Release Assay

A commercial interferon-γ (IFN-γ) release assay QFT-GIT (Cellestis, Carnegie, Australia) was performed according to the manufacturer’s instructions by experienced medical technologists. Test results were interpreted according to the cut-off values provided by the manufacturer.

### Statistical Analyses

Statistical analyses were performed using SPSS version 17.0 (SPSS Inc., Chicago, IL, USA). Goodness-of-fit χ^2^ tests were used to evaluate Hardy–Weinberg equilibrium (HWE) in the healthy subjects. Age and gender were compared between study groups using the independent *t*-test, χ^2^ test or one-way ANOVA. Three genetic models (additive, dominant, and recessive) were used to assess the genotype distribution difference between cases and controls by multivariate logistic regression with age and gender as covariates. Detailed descriptions of these three genetic models are shown in the Supplementary Materials. We calculated the power of our study design using Power and Sample Size Calculation software.^2^ We used the Haploview software package (version 4.2) for analyzing the LD patterns. A multiple-SNP score analysis was also performed as described previously (23), in which we evaluated the trend for association according to the number of relevant alleles the subjects carried. We used Bonferroni correction to adjust the *P*-values. A two-sided value of *P* < 0.0071 (0.05/7) was considered statistically significant.

## Results

### Demographic Data

The demographic characteristics in each cohort are shown in Table 1. There were no significant differences between cases and controls except for age in CLC. The TB patients in CLC were younger than LTBI individuals or HCs.

**Table 1:** Characteristics of the study participants.

Characteristics	ATAPC			CC			CLC			
	TB patients (*N* = 613)	Controls (*N* = 603)	*P*	TB patients (*N* = 580)	Controls (*N* = 605)	*P*	TB patients(*N* = 208)	LTBI individuals (*N* = 200)	Healthy controls (*N* = 204)	*P*
Age mean ± SD, years	34.5 ± 14.5	34.6 ± 13.8	0.909^a^	36.6 ± 15.6	37.2 ± 15.7	0.506^a^	38.8 ± 17.0	49.1 ± 15.9	45.7 ± 14.9	<0.001^b^
Male, *n* (%)	392 (63.9)	404 (67.0)	0.263^c^	295 (50.9)	301 (49.8)	0.702^c^	107 (51.2)	95 (47.5)	93 (45.6)	0.510^c^

*ATAPC, Aba Tibetan Autonomous Prefecture cohort; CC, Chengdu cohort; CLC, Chengdu latent tuberculosis infection cohort; TB, tuberculosis; LTBI, latent tuberculosis infection*.

*^a^Calculated by independent sample t-test*.

*^b^Calculated by one-way ANOVA*.

*^c^Calculated by χ^2^ test*.

### Genotyping Results and Distribution in Each Group

The genotype call rates of all SNPs were 99.99% and the consistency of repeat genotyping results for 5% of the samples was 100%. The SNP location, functional consequence, and the MAF in the controls of each cohort as well as Chinese Han in Beijing based on the 1000 Genomes Project database are presented in Table 2. Five SNPs are located in exons (four missense and one synonymous) as well as an upstream variant (rs10004195) and an intron variant (rs11466617). The MAFs of these SNPs in each cohort were similar to those in the 1000 Genomes Project database. The genotype distributions in each cohort are shown in Table 3. All the SNP genotype distributions were consistent with HWE in controls.

**Table 2:** Description of selected tagSNPs.

SNP	Location^a^	Functional consequence	Minor allele	Minor allele frequency			
				ATAPC	CC	CLC	1000 Genomes
rs10004195	Chr4:38,783,103	Upstream	A	0.483	0.534	0.461	0.597
rs11466617	Chr4:38,778,850	Intron	C	0.314	0.396	0.353	0.432
rs11096957	Chr4:38,774,870	Missense	T	0.389	0.409	0.475	0.373
rs11466653	Chr4:38,774,614	Missense	G	0.110	0.090	0.064	0.092
rs11096956	Chr4:38,774,559	Synonymous	A	0.414	0.483	0.417	0.524
rs11466655	Chr4:38,774,449	Missense	T	0.170	0.207	0.243	0.218
rs4129009	Chr4:38,773,268	Missense	C	0.268	0.340	0.319	0.364

*SNP, single-nucleotide polymorphism; ATAPC, Aba Tibetan Autonomous Prefecture cohort; CC, Chengdu cohort; CLC, Chengdu latent tuberculosis infection cohort*.

*^a^GRCh38.p7*.

**Table 3:** Genotype distributions in ATAPC, CC, and CLC.

SNP	Genotype	ATAPC			CC			CLC			
		TB patients, *n*(%)	Controls, *n*(%)	*P*_HWE_	TB patients, *n*(%)	Controls, *n*(%)	*P*_HWE_	TB patients, *n*(%)	LTBI individuals, *n*(%)	Healthy controls, *n*(%)	*P*_HWE_
rs10004195	TT	193 (31.5)	158 (26.2)	0.663	158 (27.2)	136 (22.5)	0.457	64 (30.6)	45 (22.5)	50 (24.5)	0.090
	TA	290 (47.3)	306 (50.8)		273 (47.1)	292 (48.3)		105 (50.2)	99 (49.5)	120 (58.8)	
	AA	130 (21.2)	138 (22.9)		149 (25.7)	177 (29.3)		40 (19.1)	56 (28.0)	34 (16.7)	
rs11096956	CC	242 (39.5)	210 (34.8)	0.642	183 (31.6)	166 (27.4)	0.508	72 (34.4)	58 (29.0)	63 (30.9)	0.449
	CA	288 (47.0)	287 (47.6)		281 (48.4)	294 (48.6)		109 (52.2)	95 (47.5)	112 (54.9)	
	AA	83 (13.5)	106 (17.6)		116 (20.0)	145 (24.0)		28 (13.4)	47 (23.5)	29 (14.2)	
rs11466617	TT	353 (57.6)	292 (48.5)	0.101	244 (42.1)	221 (36.5)	0.974	90 (43.1)	76 (38.0)	77 (32.8)	0.539
	TC	228 (37.2)	242 (40.2)		253 (43.6)	289 (47.8)		102 (48.8)	89 (44.5)	100 (53.9)	
	CC	32 (5.2)	68 (11.3)		83 (14.3)	95 (15.7)		17 (8.1)	35 (17.5)	27 (13.2)	
rs11466655	CC	426 (69.5)	408 (67.7)	0.189	337 (58.1)	383 (63.3)	0.463	120 (57.4)	131 (65.5)	117 (57.4)	0.488
	CT	173 (28.2)	185 (30.7)		217 (37.4)	193 (31.9)		75 (35.4)	59 (29.5)	75 (36.8)	
	TT	14 (2.3)	10 (1.7)		26 (4.5)	29 (4.8)		15 (7.2)	10 (5.0)	12 (5.9)	
rs4129009	TT	375 (61.2)	323 (53.7)	0.889	279 (48.1)	261 (43.1)	0.698	103 (49.3)	96 (48.0)	87 (42.6)	0.337
	TC	212 (34.6)	235 (39.0)		243 (41.9)	276 (45.6)		92 (44.0)	80 (40.0)	104 (51.0)	
	CC	26 (4.2)	44 (7.3)		58 (10.0)	68 (11.2)		14 (6.7)	24 (12.0)	13 (6.4)	
rs11096957	GG	203 (33.1)	215 (35.7)	0.080	168 (29)	213 (35.2)	0.786	54 (25.8)	71 (35.5)	54 (26.5)	0.701
	GT	317 (51.7)	307 (50.9)		298 (51.4)	289 (47.8)		107 (51.2)	90 (45.0)	106 (52.0)	
	TT	93 (15.2)	81 (13.4)		114 (19.7)	103 (17.0)		48 (23.0)	39 (19.5)	44 (21.6)	
rs11466653	AA	457 (74.6)	476 (78.9)	0.580	486 (83.8)	500 (82.6)	0.652	179 (85.6)	168 (84.0)	179 (87.7)	0.576
	AG	144 (23.5)	121 (20.1)		89 (15.3)	101 (16.7)		29 (13.9)	30 (15.0)	24 (11.8)	
	GG	12(2.0)	6 (1.0)		5 (0.9)	4 (0.7)		1 (0.5)	2 (1.0)	1 (0.5)	

*SNP, single-nucleotide polymorphism; ATAPC, Aba Tibetan Autonomous Prefecture cohort; CC, Chengdu cohort; CLC, Chengdu latent tuberculosis infection cohort; TB, tuberculosis; LTBI, latent tuberculosis infection; HWE, Hardy–Weinberg equilibrium*.

*P_HWE_ were calculated using goodness-of-fit χ^2^ tests in controls*.

### Analyses of Single *TLR10* tagSNPs and Tuberculosis (TB) Risk

#### Association Analysis in Aba Tibetan Autonomous Prefecture Cohort

As shown in Table 4, two SNPs (rs11466617 and rs4129009) showed statistically significant differences after Bonferroni correction (*P* < 0.0071) in Tibetans by comparing the genotype distributions of TB patients with HCs. The rs11466617 variant was associated with decreased susceptibility to TB under three genetic models (additive: *P* = 4.2 × 10^−5^, OR = 0.691, 95% CI = 0.578–0.826; dominant: *P* = 2.3 × 10^−4^, OR = 0.696, 95% CI = 0.555–0.873; recessive: *P* = 0.003, OR = 0.434, 95% CI = 0.280–0.671). Likewise, rs4129009 had a higher MAF in the HCs in comparison with the TB patient group (additive: *P* = 0.002, OR = 0.748, 95% CI = 0.622–0.903). In contrast, no significant differences were found for the other five SNPs under any models.

**Table 4:** Association between *TLR10* tagSNPs and risk of TB in ATAPC.

SNP	Additive model		Dominant model		Recessive model	
	OR (95% CI)	*P*	OR (95% CI)	*P*	OR (95% CI)	*P*
rs10004195	0.870 (0.742–1.020)	0.086	0.772 (0.602–0.991)	0.042	0.905 (0.689–1.187)	0.470
rs11096956	0.836 (0.711–0.985)	0.032	0.825 (0.653–1.042)	0.106	0.732 (0.536–1.001)	0.051
rs11466617	0.691 (0.578–0.826)	**4.86 × 10^−5^**	0.696 (0.555–0.873)	**0.002**	0.434 (0.280–0.671)	**1.77 × 10^−4^**
rs11466655	0.956 (0.767–1.191)	0.686	0.920 (0.722–1.173)	0.501	1.372 (0.604–3.116)	0.449
rs4129009	0.748 (0.622–0.903)	**0.002**	0.735 (0.619–0.902)	0.008	0.565 (0.343–0.931)	0.025
rs11096957	1.100 (0.930–1.302)	0.267	1.123 (0.886–1.424)	0.337	1.144 (0.829–1.578)	0.414
rs11466653	1.287 (1.008–1.643)	0.043	1.285 (0.983–1.678)	0.066	1.977 (0.737–5.306)	0.176

*P-values significant after Bonferroni correction (P < 0.0071) are shown in bold*.

*SNP, single-nucleotide polymorphism; OR, odds ratio; CI, confidence interval*.

*P-values were calculated using multivariate logistic regression with age and gender set as covariates*.

#### Association Analysis in Chengdu Cohort

The analyses of association between *TLR10* SNPs and TB in CC are shown in Table 5. We tested the same SNPs, with the aim of enhancing the reliability of the associations by attempting to replicate them in different populations. Five SNPs showed a value of *P* < 0.05 under some of the genetic models, e.g., rs10004195 (additive: *P* = 0.043), rs11096956 (additive: *P* = 0.049), rs11466617 (additive: *P* = 0.049), and rs11096957 (additive: *P* = 0.027, dominant: *P* = 0.021) However, we did not find any statistically significant locus related to an individual’s susceptibility to TB after Bonferroni correction.

**Table 5:** Association between *TLR10* tagSNPs and risk of TB in CC.

SNP	Additive model		Dominant model		Recessive model	
	OR (95% CI)	*P*	OR (95% CI)	*P*	OR (95% CI)	*P*
rs10004195	0.849 (0.725–0.995)	0.043	0.770 (0.591–1.004)	0.053	0.833 (0.645–1.076)	0.162
rs11096956	0.852 (0.725–1.000)	0.049	0.819 (0.637–1.052)	0.118	0.791 (0.600–1.043)	0.097
rs11466617	0.865 (0.734–1.019)	0.083	0.790 (0.625–0.999)	0.049	0.896 (0.651–1.233)	0.499
rs11466655	1.159 (0.952–1.410)	0.141	1.247 (0.987–1.575)	0.064	0.941 (0.547–1.620)	0.827
rs4129009	0.868 (0.730–1.031)	0.107	0.818 (0.651–1.029)	0.086	0.876 (0.605–1.269)	0.483
rs11096957	1.203 (1.021–1.418)	0.027	1.335 (1.045–1.707)	0.021	1.195 (0.890–1.605)	0.237
rs11466653	0.943 (0.709–1.254)	0.684	0.921 (0.679–1.250)	0.598	1.308 (0.349–4.899)	0.691

*SNP, single-nucleotide polymorphism; OR, odds ratio; CI, confidence interval*.

*P-values were calculated using multivariate logistic regression with age and gender set as covariates*.

#### Association Analysis in Chengdu LTBI Cohort

*Due to a poor verification* in the CC, we further investigated the association between *TLR10* SNPs and TB risk by comparing the genotype distributions in the TB-uninfected group, the LTBI group, and the active TB group. We noted that the rs11466617 CC genotype exhibited a significantly higher frequency in the LTBI group, as compared with the active TB patients under the recessive model (shown in Table 6; *P* = 0.004, OR = 0.389, 95% CI = 0.204–0.744). There were no other SNPs that showed significant differences after Bonferroni correction under any genetic model either in comparison of the TB-uninfected with LTBI individuals or in comparison of LTBI individuals with active TB patients.

**Table 6:** Association between *TLR10* tagSNPs and risk of TB and latent infection in the Chengdu latent tuberculosis infection cohort.

SNP	Model	LTBI individuals vs. healthy controls		TB patients vs. LTBI individuals	
		OR (95% CI)	*P*	OR (95% CI)	*P*
rs10004195	Additive	1.355 (1.010–1.818)	0.043	0.706 (0.526–0.947)	0.020
	Dominant	1.155 (0.725–1.841)	0.543	0.636 (0.398–1.016)	0.058
	Recessive	1.929 (1.190–3.128)	0.008	0.622 (0.382–1.015)	0.058
rs11096956	Additive	1.276 (0.958–1.700)	0.096	0.721 (0.535–0.970)	0.031
	Dominant	1.091 (0.709–1.677)	0.692	0.782 (0.503–1.216)	0.275
	Recessive	1,908 (1.139–3.196)	0.014	0.494 (0.287–0.849)	0.011
rs11466617	Additive	1.111 (0.836–1.476)	0.468	0.707 (0.520–0.961)	0.027
	Dominant	0.978 (0.651–1.468)	0.913	0.796 (0.524–1.210)	0.286
	Recessive	1.483 (0.854–2.576)	0.162	0.389 (0.204–0.744)	**0.004**
rs11466655	Additive	0.779 (0.559–1.086)	0.141	1.295 (0.922–1.819)	0.136
	Dominant	0.712 (0.475–1.067)	0.100	1.357 (0.892–2.066)	0.154
	Recessive	0.858 (0.360–2.044)	0.729	1.482 (0.618–3.553)	0.378
rs4129009	Additive	1.018 (0.749–1.383)	0.187	0.866 (0.732–1.187)	0.370
	Dominant	0.809 (0.544–1.204)	0.296	0.990 (0.656–1.494)	0.963
	Recessive	2.081 (1.021–4.241)	0.044	0.495 (0.240–1.023)	0.057
rs11096957	Additive	0.798 (0.604–1.054)	0.112	1.290 (0.967–1.723)	0.084
	Dominant	0.653 (0.425–1.001)	0.051	1.578 (1.010–2.468)	0.045
	Recessive	0.870 (0.535–1.416)	0.576	1.216 (0.736–2.009)	0.446
rs11466653	Additive	1.338 (0.789–2.270)	0.280	0.905 (0.529–1.547)	0.715
	Dominant	1.348 (0.764–2.378)	0.303	0.922 (0.520–1.634)	0.781
	Recessive	1.960 (0.172–22.28)	0.587	0.518 (0.037–7.263)	0.625

*P-values significant after Bonferroni correction (P < 0.0071) are shown in bold*.

*TB, tuberculosis; LTBI, latent tuberculosis infection*.

*P-values were calculated using multivariate logistic regression with age and gender set as covariates*.

### Analyses of Multiple-SNP Score on TB Risk

**Figure 1:**
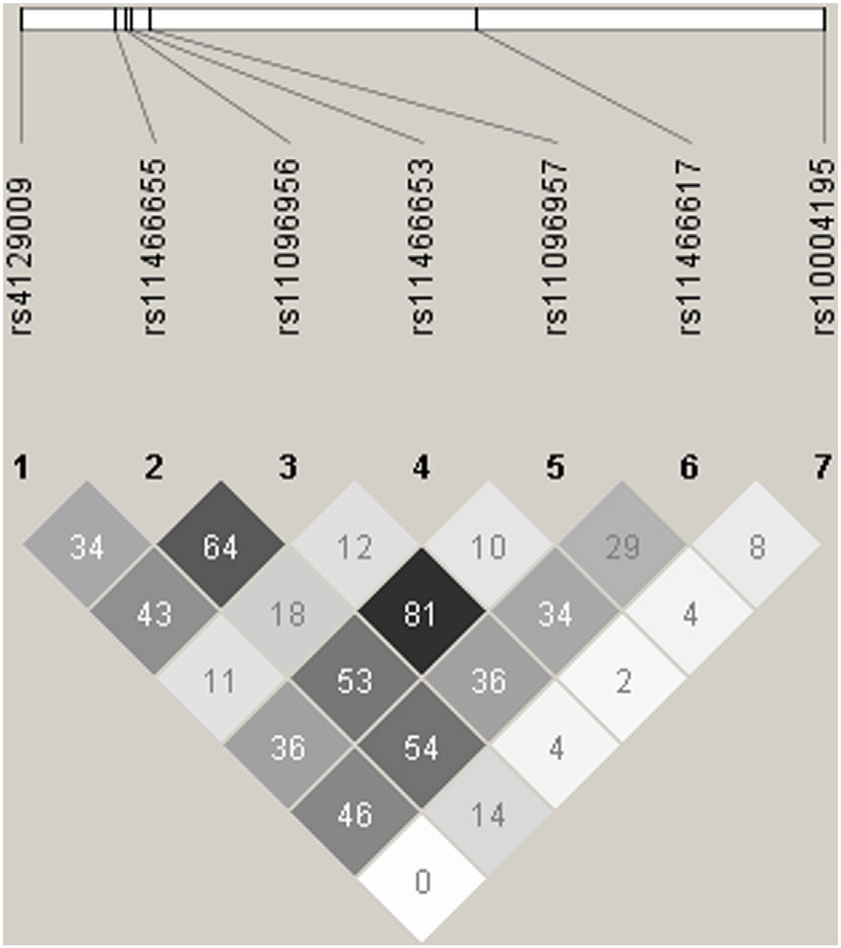
Linkage disequilibrium (LD) plots for *LTR10*. The LD plots were generated by Haploview 4.2. Polymorphisms are identified by their dbSNP rs numbers, and their relative positions are marked by vertical lines within the white horizontal bar. The numbers within squares indicate the *r*^2^ value, expressed as a percentile.

Given that the *TLR10* gene variants were shown to have a dose-dependent effect on cytokine production, we also performed a multiple-SNP score analysis on TB risk in the three case–control studies. We first pooled the subjects from ATAPC and CC together and obtained four SNPs (rs10004195 rs11096956, rs11466617, rs4129009) with a significant difference (*P* < 0.0071, data not shown). As the LD between these tagSNPs was low (*r*^2^ < 0.5, as shown in Figure 1), we hypothesized that these polymorphisms influence susceptibility to TB risk independently of one another. As shown in Table 7, there was a correlation between the number of *TLR10* protective alleles carried and decreasing TB risk in the analysis in ATAPC (*P* = 0.002 and 1.94 × 10^−4^ for trend). However, this dose-dependent protective effect was not observed in CC or CLC.

**Table 7:** Multi-SNP analysis of rs10004195, rs11096956, rs11466617, and rs4129009 in all cohorts.

Number of protective alleles carried	Odds ratios			
	ATAPC	CC	CLC	
			LTBI individuals vs. healthy controls	TB patients vs. LTBI individuals
0	1	1	1	1
1	1.143	0.814	1.720	0.700
2	1.179	0.895	1.157	0.891
3	0.715	0.856	2.396	0.403
4	0.702	0.807	1.230	0.724

Trend *P*-value	**0.002**	0.040	0.176	0.028

*P-values significant after Bonferroni correction (P < 0.0071) are shown in bold*.

*Trend tests were performed for the increasing number of *TLR10* protective alleles (0, 1, 2, 3, or 4) carried and decreasing odds of disease susceptibility for each cohort. ATAPC, Aba Tibetan Autonomous Prefecture cohort; CC, Chengdu cohort; CLC, Chengdu latent tuberculosis infection cohort; TB, tuberculosis; LTBI, latent tuberculosis infection*.

### Power Analysis

In order to assess whether the power of study with the current simple size was sufficient, we calculated the power of all the SNPs under an allelic model. As shown in Figure S1 in Supplementary Material, the frequency of the SNPs in each group was sufficient to provide >90% power to detect a common risk allele with an OR of 1.6, except for rs11466653 in CLC.

## Discussion

Strong evidence supports a critical role for genetic factors in susceptibility to TB infection and progression (24). In this self-validating case–control association study, we used tagSNPs that captured all the common variations in *TLR10* to investigate the role of genetic variation in susceptibility to TB. We found two tagSNPs (rs11466617 and rs4129009) that were associated with TB susceptibility, most notably the C-allele of rs11466617 that exhibited a strong association with decreased TB risk in the Tibetan population. However, these two associations were not validated in the study of the Chinese Han population. We also observed that the CC genotype of rs11466617 was associated with resistance to activation from LTBI, but not associated with TB infection in the Han Chinese population. Additionally, we identified a dose-dependent protective effect in TB susceptibility among individuals carrying the “protective” alleles of *TLR10*.

Toll-like receptor family members play critical roles in the defense against Mtb infection, especially TLR2 that can trigger an immune response against Mtb by recognizing Mtb components and inducing pro-inflammatory signals (25), including the MyD88- and TRIF-dependent pathway (26, 27). However, very few functional studies have implicated TLR10 in the response to Mtb infection either *in vivo* or *in vitro*. Evidence shows that TLR10 in cooperation with TLR2 can recruit the proximal adaptor MyD88 to the complex, but fails to trigger the MyD88- and TRIF-dependent signaling, suggesting TLR10 functions as a negative regulator (15, 28), which may be involved in the progression of TB. Furthermore, this inhibitory property of TLR10 in immune regulation upon stimulation with TLR2 ligands is affected by *TLR10* polymorphisms (14). We found that two SNPs of *TLR10* were associated with decreased risk of TB in the Tibetan but not in the Chinese Han population, suggesting racial differences may impact on the risk of TB associated with these SNPs. Another underlying reason to explain this inconsistent result could be environmental factors. In particular, the Tibetans recruited in the present study lived in a region with higher altitude, which is considered to be a risk factor for TB (29). Therefore, a proportion of subjects in ATAPC may not suffer active TB disease if living in the region where the CC was conducted. Moreover, we used Bonferroni correction, in other words regarded a *P* < 0.0071 as the threshold for significance, to limit the type-1 error. However, reducing the chance of a type-1 error in this way may increase the possibility of a type-2 error. In the present study, we observed that a few SNPs showed a borderline *P*-value in CC, including rs11466617, the most significant locus in ATAPC.

It is widely accepted that the pathogenesis of TB can be thought of as a two-stage process: LTBI, the establishment of a productive infection but remaining asymptomatic, and active TB disease which is characterized by growth of Mtb in sputum and cultivation in culture or positive AFB smear, plus characteristic radiological signs and hallmark symptoms (30). Early observations detected considerable interindividual variability in the establishment of TB infection after exposure to Mtb in sailors who shared similar known environmental factors, suggesting genetic factors may influence this stage (31). On the other hand, only 5–15% of infected individuals develop active TB, indicating that genetic factors are associated with an individual’s susceptibility to trigger an active TB disease (2). However, the mechanisms involved in the host–pathogen interaction in each stage are extremely complex and not fully understood (32). It is reasonable to assume that different genetic polymorphisms may influence different stages. Given this hypothesis, we performed an association study in CLC in which we distinguished the different stages of TB, to investigate the role of *TLR10* SNPs in the pathogenesis of TB, and to further explore the inconsistent results observed in CC. We demonstrated that rs11466617 only affected the status of Mtb-infected individuals with respect to LTBI vs. active TB. The mechanism underlying this association may partly relate to the mediator function of TLR10 in macrophages after Mtb has been engulfed (33), as well as a potential mechanism of apoptosis by TLR2 signaling (34). It can also explain the inconsistent result we observed in ATAPC and CC: more uninfected individuals in the control group of CC may have led to the negative result in this cohort, while the potentially higher frequency of LTBI in control group of ATAPC, based on the higher TB prevalence in Tibetan (29), may partly contribute to the significant result in ATAPC.

As the evidence from a functional study of *TLR10* showed gene variant dosage effects on cytokine production (14), we also carried out a multi-SNP score test in three cohorts. We found that the *TLR10* SNPs showed a dose-dependent protective effect on TB risk in Tibetans. Therefore, further study investigating the mechanism of *TLR10* in the pathogenesis of TB could build on this observation.

Studies exploring the functional effects of *TLR10* SNPs and their role in susceptibility to various diseases have been increasing. We searched the PubMed database using the key words “polymorphism” “SNP” “mutation” “variation” combined with “TLR10.” By screening of abstracts and full texts, we found a total of 56 relevant studies that investigated *TLR10* SNPs in relation to phenotypes including infectious disease, carcinoma, allergic, and autoimmune disease as well as altered immunoreactions *in vitro* (see Table S2 in Supplementary Material). We also noted that three studies, using various designs, demonstrated that *TLR10* SNPs were associated with susceptibility to TB. Bulat-Kardum et al. found that the rs11096957 AA genotype was associated with a predisposition to TB in the Caucasian population (8). However, in the present study, we did not observe association between rs11096957 and risk of TB. This inconsistent result is likely due to the ethnic difference, as the frequency of the A allele was much higher in the Caucasian population (0.6) than in the Han Chinese population (0.37), based on the 1000 Genomes Project data. Ma et al. demonstrated that nonsynonymous polymorphisms of *TLR10* were significantly associated with TB in African and European Americans (18). The present study identified that rs4129009, which causes an amino-acid change in the TIR domain of the intracellular portion of the protein and results in increased cytokine responses (35), showed decreased risk of TB in Tibetans. Moreover, Uren et al. observed that an SNP (rs12233670) upstream of *TLR10* was associated with TB risk by using genome-wide association data and case–control analysis of South Africans (36). Although we did not genotype this SNP, another upstream SNP (rs10004195) that is in perfect LD (*r*^2^ = 1) with rs12233670 was tested in our study. However, this locus was not associated with risk of TB in present study. Differences in ethnicity and study design may explain the inconsistent result.

Our study has several strengths. (a) This investigation represents the first exploration of the association between *TLR10* genetic variants and TB risk in a Chinese population, with two ethnic groups and an additional study of TB infection status. In this study, we used the genetic database of Chinese Beijing Han to conduct tagSNP selection, as Chinese Han and Tibetans share a similar genetic background (37). The SNPs we studied cover the vast majority of the functional SNPs of *TLR10* investigated in previous studies (Table S2 in Supplementary Material). (b) The sample size in the present study was sufficient to provide >90% power to detect a common risk allele with an OR of 1.6, except for rs11466653 in CLC (see Figure S1 in Supplementary Material). (c) Furthermore, in order to avoid the possibility of obtaining a false positive result, we used Bonferroni correction to adjust the *P*-values. We also limited potential confounding factors by excluding coinfection with HIV, comorbidities such as cancer, diabetes, immune system diseases, and chronic respiratory diseases as well as steroid use for inflammatory conditions.

Some limitations in this study must be noted. First, rs11466617 and rs4129009 were associated with decreased risk of TB in Tibetans but these associations did not replicate in the verification cohort of Chinese Han. Second, we did not elucidate the mechanism of how these variations affect the host immune response and the progression of TB in the subjects, although we noted that rs11466617 may only affect an individual’s susceptibility to progress from LTBI to active TB disease. Third, there is no gold standard diagnostic test for LTBI to date, though INF-γ release assay has an acceptable specificity, positive and negative predictive values for LTBI detection (38).

## Conclusion

In summary, the present study indicated that two SNPs (rs11466617 and rs4129009) as well as a dose-dependent effect of *TLR10* SNPs were associated with a decreased susceptibility to TB in Tibetans, but not in Chinese Han. Moreover, individuals carrying the rs11466617 CC genotype showed decreased risk of progression to active TB from LTBI. However, no genotype of *TLR10* was associated with the establishment of LTBI after Mtb exposure. More studies are required to validate these findings in different populations and to elucidate the mechanistic role of *TLR10* variants in TB susceptibility.

## Ethics Statement

All protocols for this study were reviewed and approved by the Institutional Review Board of the West China Hospital of Sichuan University in 2013 (Protocol no. 131). Written informed consent was obtained from all the study participants.

## Author Contributions

YW, M-MZ, and W-WH carried out the molecular genetic studies, statistical analysis, and drafted the manuscript. AS and J-QH contributed to the study conception and design, analysis, and interpretation of the data, and helped revise the manuscript. S-QW, M-GW, and X-yT carried out the sample acquisition and genotyping assays.

## Conflict of Interest Statement

The authors declare that the research was conducted in the absence of any commercial or financial relationships that could be construed as a potential conflict of interest.

## Footnotes

^1^http://www.hapmap.org.

^2^http://biostat.mc.vanderbilt.edu/PowerSampleSize.

## Supplementary Material

Figure S1 Statistical power of each SNP in the ATAPC **(A)**, CC **(B)**, and CLC **(C)** cohorts. Power was calculated by the Power and Sample Size Calculation software.
