# Supplementary figures and images for: Polymorphisms in Toll-Like Receptor 10 and Tuberculosis Susceptibility: Evidence from Three Independent Series
Source: Front Immunol. 2018 Feb 23;9:309. doi: 10.3389/fimmu.2018.00309 (PMC5829065; doi:10.3389/fimmu.2018.00309)

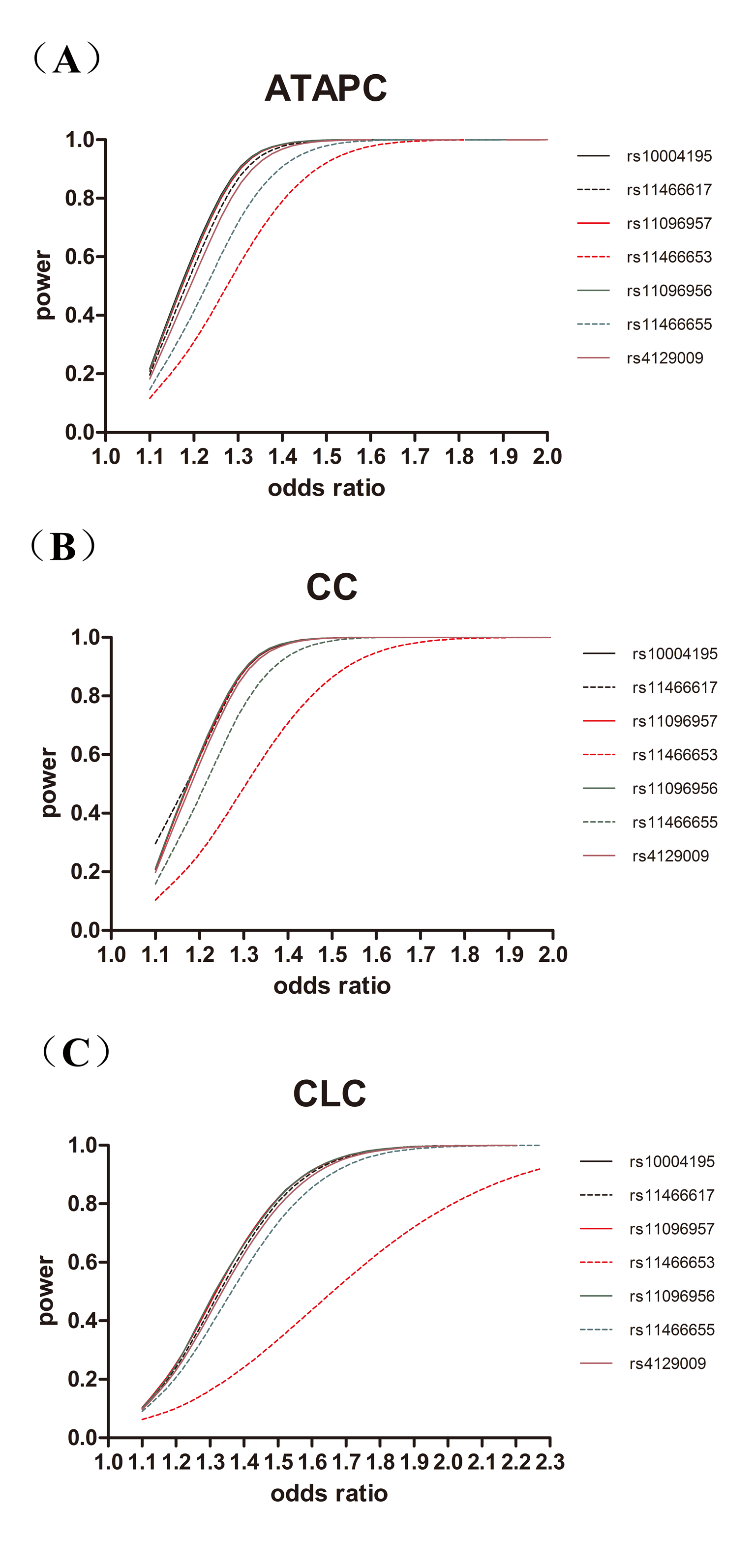

Supplement: Figure S1 — Statistical power of each SNP in the ATAPC (A), CC (B), and CLC (C) cohorts. Power was calculated by the Power and Sample Size Calculation software. [file image_1.tif]
