# Supplementary material for: Polymorphisms in Toll-Like Receptor 10 and Tuberculosis Susceptibility: Evidence from Three Independent Series
Source: Front Immunol. 2018 Feb 23;9:309. doi: 10.3389/fimmu.2018.00309 (PMC5829065; doi:10.3389/fimmu.2018.00309)
Supplement: Supplementary file 2 [file presentation_1.PDF]

Polymorphisms in Toll-like receptor 10 and tuberculosis susceptibility: evidence from three independent series

Yu Wang *et al*

Supplementary Materials

1. SNPscan genotyping assays

100-200 ng of DNA was first denatured at 98 ℃ for 5 min in a10 μl reaction containing 1x DNA lysis buffer, and then mixed well with a 10 μl ligation premix composed of 2 μl 10x Ligase buffer, 0.5 μl Ligase, 1 μl Probe Mix and 7.5 μl Mili-Q water. The ligation reaction was performed on a ABI 2720 thermal cycler under the following cycling program: 4 cycles of (94 ℃ 1 min, 58 ℃ 4 hr), 94 ℃ 2 min, hold at 4 ℃ and immediately stopped by adding 20 μl of 2x Stop Buffer. Two 48-plex fluorescence PCR reactions were performed for each ligation product. Each PCR reaction was prepared in a 20 μl mixture containing 1x PCR Master Mix, 1 μl Primer Mix Set A or Set B and 1 μl ligation product. The PCR program was as follows: 95 ℃ 2 min; 9 cycles of 94 ℃ 20s, 65 ℃ - 0.5 ℃ /cycle 40s, 72 ℃ 1.5 min; 25 cycles of 94 ℃ 20 s, 57 ℃ 40 s, 72 ℃ 1.5 min; 60 ℃ 1 hr; hold at 4 ℃. PCR products were separated and detected by capillary electrophoresis on an ABI3730XL sequencer. Raw data were analyzed by GeneMapper 4.0 and genotypes for each locus were determined based on the information of the allele specific ligation-PCR product's labeling dye color and fragment size.

2. Genetic models used to assess genotype distributions

a) Additive: this is a general model in which individuals carrying one copy of the variant allele are at increased risk of disease and individuals carrying two copies are at 2-fold higher relative risk.

b) Dominant: for this model, the presence of a single copy of the variant allele is sufficient to account for disease susceptibility. The presence of the second variant allele does not confer any additional disease risk over and above that of the first allele.

c) Recessive: the recessive model requires both copies of the variant allele to be present in a given individual in order to confer increased risk of the disease. Individuals carrying only one copy of the variant allele are phenotypically indistinguishable from wild-type individuals.

3. Supplementary Table 1: Inclusion and exclusion criteria of each cohort.

Inclusion criteria		
ATAPC	TB patient group	1) Clinical TB diagnosis of subjects was based on the criteria of the U.S. Department of Health & Human Services (https://www.cdc.gov/tb/publications/factsheets/testing/diagnosis.pdf), including

		5 points: medical history, physical examination, test for TB infection (tuberculin skin test or QuantiFERON TB-GIT assay), chest radiograph, diagnostic microbiology. The diagnosis of TB was conducted by two senior TB specialists (JQH and XYT).<br>2) self-identified Tibetan.<br>3) age > 14 years.<br>4) long-term resident in Aba Tibetan Autonomous Prefecture.<br>5) the diagnosis time was between February 2013 and August 2015.
	control group	1) individuals who had undergone routine health examination in the People's Hospital of Aba Tibetan Autonomous Prefecture during the same period.<br>2) gender and age matched to the cases.<br>3) self-identified Tibetan.<br>4) unrelated to the TB patients<br>5) normal chest radiograph.<br>6) without TB history.
CC	TB patient group	1) Clinical TB diagnosis with subjects was based on the criteria of the U.S. Department of Health & Human Services (https://www.cdc.gov/tb/publications/factsheets/testing/diagnosis.pdf), including 5 points: medical history, physical examination, test for TB infection (tuberculin skin test or QuantiFERON TB-GIT assay), chest radiograph, diagnostic microbiology. The diagnosis of TB was conducted by two senior TB specialists (JQH and XYT).<br>2) self-identified Chinese Han.<br>3) age > 14 years.<br>4) long-term resident in Chengdu.<br>5) the diagnosis time was between July 2012 and August 2014.
	control group	1) individuals who had undergone routine health examination in the People's Hospital of Aba Tibetan Autonomous Prefecture during the same period.<br>2) gender and age matched to the cases.<br>3) self-identified Chinese Han.<br>4) unrelated to the TB patients.<br>5) normal chest radiograph.<br>6) without TB history.
CLC	TB patient group	1) bacteriology-confirmed cases having positive of culture and/or acid-fast bacilli and/or polymerase chain reaction of Mtb DNA in sputum or body sites, with a typical clinical complaint, e.g. fever, cough, sweating, hemoptysis, or weight loss.<br>2) self-identified Chinese Han.<br>3) the diagnosis time was between June 2014 and December 2015.
	LTBI group	1) contacts of TB patients.<br>2) positive result for the QuantiFERON TB-GIT assay.<br>3) without any clinical symptom.<br>4) self-identified Chinese Han.<br>5) unrelated to the TB patients and healthy controls.<br>6) with normal chest radiograph.<br>7) without TB history.<br>8) recruited during the same period.
	Healthy control group	1) contacts of TB patients.<br>2) having negative result for the QuantiFERON TB-GIT assay.<br>3) without any clinical symptom.<br>4) self-identified Chinese Han.<br>5) unrelated to the LTBI individuals and healthy controls.<br>6) normal chest radiograph.<br>7) without TB history.<br>8) recruited during the same period.
Exclusion criteria		
ATAPC		1) co-infection with human immunodeficiency virus.

CC	2) complication with cancer, diabetes, immune system diseases, chronic respiratory diseases, steroid use for inflammatory conditions.<br>3) Usage of steroids.
CLC	

ATAPC, Aba Tibetan Autonomous Prefecture cohort; CC, Chengdu cohort; CLC, Chengdu latent tuberculosis infection cohort; TB, tuberculosis.

3. Supplementary Table 2: studies of *TLR10* SNPs in relation to phenotype.

**Study and years**	**SNP(s)**	**Research subjects / cell types**	**Associated phenotype**
Tongtawee et al. 2017	rs10004195	Patients with chronic abdominal pain	Gastric mucosal patterns and inflammation
Törmänen et al. 2017	rs4129009	Asthmatic patients	Need for inhaled corticosteroids and persistent asthma
de Barros Gallo et al. 2017	rs11096957	Patients with oral cancer and malignant disorder	No association was found
Törmänen et al. 2017	rs4129009	Asthmatic patients	Asthma in preschool-aged children after infant bronchiolitis
Caitlin et al. 2017	rs12233670	Patients with tuberculosis	Tuberculosis
Tongtawee et al. 2017	rs10004195	Patients with chronic abdominal pain.	*H. pylori* infection
Henmyr et al. 2017	Genome-wide sequencing	Allergic rhinitis patients	Excess of rare variants was associated with allergic rhinitis
Smith et al. 2017	rs11466645, rs11466617, rs11725309	Agricultural workers exposed to organic dust	Increased IL-6 and TNF-α level in serum
Nishikawa et al. 2016	rs11096957	Patients with chronic plaque psoriasis	treatment response to anti-TNF-a biologic therapy
Nuolivirta et al. 2016	rs4129009	Infants with bronchiolitis	Increased serum IgE
Lauhkonen et al. 2016	rs4129009	Children with bronchiolitis	No association was found
Ravishankar et al. 2015	rs10004195	Patients with upper gastrointestinal disorder	*H. pylori* infection-dependent gastroduodenal disease
Traks et al. 2015	rs10776482, rs7694115, rs7660429	Vitiligo patients	Vitiligo
Ammerdorffer et al. 2016	rs11096957, rs4129009, rs11096955,	Chronic Q fever patients and PBMCs	Inhibition of cytokine responses, but not with Chronic Q fever
Sung et al. 2015	rs10004195	Male smokers	Anti-*H. pylori* antibody levels and persistence of infection
Stappers et al. 2015	rs4129009, rs11096955, rs11096957	Patients with complicated skin and skin structure infections, PBMCs and HEK-293cells	Complicated skin and skin structure infections, and interleukin 6 secretion by PBMCs in response to pathogens
Bulat-Kardum	rs11096957	Tuberculosis	Tuberculosis

et al. 2015		patients	
Gimalova et al. 2014	rs11466617	Patients with atopic dermatitis	Atopic dermatitis
Tang et al. 2015	rs10004195	Patients with *H. pylori* infection	*H. pylori* infection
Matas-Cobos et al. 2015	rs4129009	Patients with acute pancreatitis	No association was found
Cho et al. 2015	rs4129009, rs10004195	Patients with autoimmune thyroid disease	Autoimmune thyroid disease
Oosting et al. 2014	rs11096957, rs11466653, rs11096955, rs4129009	PBMCs from healthy individuals	Production of IL-1β, IL-6, IL-8, and TNF-α when stimulated with TLR2 ligands
Xiao et al. 2015	rs4504265, rs11466655, rs11096957, rs10856839	Patients with Graves' disease	No association was found
Kim et al. 2014	rs10004195	Patients with benign prostatic hyperplasia	No association was found
Smith et al. 2014	rs10856838, rs11096955, rs11466640, rs11466652, rs4129009, rs7658893	Patients with chronic cavitary pulmonary aspergillosis	No association was found
Laayouni et al. 2014	rs11096957	PBMCs from healthy individuals	Cytokine production induced by stimulation with *Y. pestis* and *Y. pseudotuberculosis*
Haerynck et al. 2013	rs7694115, rs11466645, rs11096957, rs11096956, rs4129009	Patients with cystic fibrosis	No association was found
Requena et al. 2013	rs11096955	Patients with Meniere's disease	Meniere's disease
Mikacenic et al. 2013	rs11466640, rs11096956, rs11096957	Healthy individuals	Pam_3CSK_4 (a TLR1/2 lipopeptide agonist) induced whole blood responses
Kim et al. 2013	rs11466653	Patients with papillary thyroid carcinoma	Development of tumors
Sivula et al. 2012	rs337629	Allogenic hematopoietic stem cell transplantation recipients and the donors	Graft-versus-host disease
Guirado et al. 2012	rs4129009	Patients with urothelial bladder cell carcinoma	Stage of tumor infiltration and overall survival
Morgan et al. 2012	rs6841698, rs10024216, rs4274855, rs7658893	Patients with Crohn’s disease	Crohn’s disease
Fisher et al. 2012	rs55617325	Bovine	*Mycobacterium avium* spp paratuberculosis infection.

Veltkamp et al. 2012	rs11096955, rs7658893	Patients with sarcoidosis	Sarcoidosis
Miedema et al. 2012	rs4274855, rs10856839, rs11096956, rs11096957, rs11466652, rs4129009	Children with acute lymphoblastic leukemia and atopic disease	No association was found
Abad et al. 2011	rs6841698, rs7653908, rs7658893	Patients with Crohn's disease	Crohn's disease
Liu et al. 2011	rs4129009	Fetal cord blood	Forkhead box protein P3 mRNA expression in cord blood mononuclear cells after lipid A and peptidoglycan stimulation
Park et al. 2010	rs1004195	Children with IgA nephropathy	IgA nephropathy
Mailaparambil et al. 2010	rrs11096955	Infants born with a gestational age ≤ 28	Bronchopulmonary dysplasia
Qian et al. 2010	rs11466651	Asthmatic patients	Allergic rhinitis in asthmatic patients
Moskvina et al. 2010	rs4274855, rs10856839, rs11096957, rs11096956, rs11096955, rs11466657, rs4129009	Asthmatic children	Asthma
Etem et al. 2011	rs4129009	Patients with rheumatoid arthritis	No association was found
Rajaraman et al. 2010	genome-wide sequencing	Patients with intracranial meningioma	No association was found
Kastelijn et al. 2009	rs11096955, rs4274855, rs7658893, rs9994896, rs9999294	Lung transplant recipients	No association was found
Heinzmann et al. 2009	rs4274855, rs11096955, rs10856839	Preterm infants born below 32 gestational weeks	No association was found
Daley et al. 2009	rs11096957, rs10776483	Asthmatic patients	Atopic asthma
Purdue et al. 2009	rs4513579, rs11096956, rs10856838, rs7660429	Patients with non-Hodgkin lymphoma.	No association was found
Mailaparambil et al. 2008	rs4129009, rs11466657, rs11096955, rs11096956, rs11096957, rs10856839, rs4274855, rs11466645	Infants with severe RSV infection	No association was found
Stevens et al. 2008	rs11096955, rs11096957,	Patients with prostate cancer	Prostate cancer

Kormann et al. 2008	rs4129009	Asthmatic children & PBMCs from adult volunteers	Atopic asthma and higher level of TLR10 mRNA expression in PBMCs
Chen et al. 2007	rs11466617, rs11466640, rs4274855, rs11096957, rs11096955, rs11466657, rs4129009	Patients with prostate cancer	No association was found
Zhou et al. 2006	rs10856837, rs11466651, rs11466652, rs11466653, rs11096956, rs11096955, rs11466655	Patients with nasopharyngeal carcinoma risk	No association was found
Puthothu et al. 2006	rs4274855, rs10856839, rs11096957, rs11096956, rs11096955, rs11466657, rs4219009	Asthmatic children	No association was found
Sun et al. 2005	rs4129009, rs11096955, rs11096957, rs4274855, *LTR10* -3260C/T, *LTR10* -1692C/T	Patients with prostate cancer	Prostate cancer
Lazarus et al. 2004	rs4129009, +1031G/T	Asthmatic patients	Asthma.

Supplementary Figure 1: Statistical power of each SNP in the ATAPC (A), CC (B) and CLC (C) cohorts. Power was calculated by the Power and Sample Size Calculation software.
